# Variation at *FCGR2A* and Functionally Related Genes Is Associated with the Response to Anti-TNF Therapy in Rheumatoid Arthritis

**DOI:** 10.1371/journal.pone.0122088

**Published:** 2015-04-07

**Authors:** Gabriela Avila-Pedretti, Jesús Tornero, Antonio Fernández-Nebro, Francisco Blanco, Isidoro González-Alvaro, Juan D. Cañete, Joan Maymó, Mercedes Alperiz, Benjamín Fernández-Gutiérrez, Alex Olivé, Héctor Corominas, Alba Erra, Adrià Aterido, María López Lasanta, Raül Tortosa, Antonio Julià, Sara Marsal

**Affiliations:** 1 Vall d'Hebron Hospital Research Institute, Rheumatology Research Group. Barcelona, Spain; 2 Hospital Universitario De Guadalajara, Rheumatology Department, Guadalajara, Spain; 3 UGC Reumatología, Instituto de Investigación Biomédica en Málaga, Hospital Regional Universitario de Málaga, Universidad de Málaga, Málaga, Spain; 4 INIBIC-Hospital Universitario A Coruña, Rheumatology Department, A Coruña, Spain; 5 Hospital Universitario de La Princesa, IIS La Princesa, Rheumatology Department, Madrid, Spain; 6 Hospital Clínic de Barcelona, Rheumatology Department, Barcelona, Spain; 7 Hospital del Mar, Barcelona, Rheumatology Department, Barcelona, Spain; 8 Hospital Universitario Central de Asturias, Rheumatology Department, Oviedo, Spain; 9 Hospital Clínico San Carlos, Madrid, Rheumatology Department, Madrid, Spain; 10 Hospital Universitari Germans Trias i Pujol, Rheumatology Department, Barcelona, Spain; 11 Hospital Moisès Broggi, Rheumatology Department, Barcelona, Spain; 12 Hospital Sant Rafael, Rheumatology Department, Barcelona, Spain; University of Leicester, United Kingdom

## Abstract

**Objective:**

Anti-TNF therapies have been highly efficacious in the management of rheumatoid arthritis (RA), but 25–30% of patients do not show a significant clinical response. There is increasing evidence that genetic variation at the Fc receptor *FCGR2A* is associated with the response to anti-TNF therapy. We aimed to validate this genetic association in a patient cohort from the Spanish population, and also to identify new genes functionally related to *FCGR2A* that are also associated with anti-TNF response.

**Methods:**

A total of 348 RA patients treated with an anti-TNF therapy were included and genotyped for *FCGR2A* polymorphism rs1081274. Response to therapy was determined at 12 weeks, and was tested for association globally and independently for each anti-TNF drug (infliximab, etanercept and adalimumab). Using gene expression profiles from macrophages obtained from synovial fluid of RA patients, we searched for genes highly correlated with *FCGR2A* expression. Tag SNPs were selected from each candidate gene and tested for association with the response to therapy.

**Results:**

We found a significant association between *FCGR2A* and the response to adalimumab (*P*=0.022). Analyzing the subset of anti-CCP positive RA patients (78%), we also found a significant association between *FCGR2A* and the response to infliximab (*P*=0.035). *DHX32* and *RGS12* were the most consistently correlated genes with *FCGR2A* expression in RA synovial fluid macrophages (*P*<0.001). We found a significant association between the genetic variation at *DHX32* (rs12356233, corrected *P*=0.019) and a nominally significant association between *RGS12* and the response to adalimumab (rs4690093, uncorrected *P*=0.040). In the anti-CCP positive group of patients, we also found a nominally significant association between *RGS12* and the response to infliximab (rs2857859, uncorrected *P*=0.042).

**Conclusions:**

In the present study we have validated the *FCGR2A* association in an independent population, and we have identified new genes associated with the response to anti-TNF therapy in RA.

## Introduction

The introduction of Tumor Necrosis Factor (TNF) inhibitors has revolutionized the treatment of rheumatoid arthritis (RA). In the clinical practice, anti-TNF alpha agents have made it possible to achieve a minimal inflammatory activity or even disease remission [1,2]. Despite their clear efficacy in RA management, there is a substantial group of patients who will fail to respond to this therapeutic approach [3]. The high costs of these therapies as well as the availability of alternative biologic therapies in RA, clearly increase the need to identify markers of response to anti-TNF agents [4].

Genetic variation has shown to influence many aspects of RA heterogeneity, including the response to anti-TNF therapy [5,6]. Genome-wide association studies (GWAS) are a powerful genetic analysis approach and have allowed the identification of new genomic regions associated with treatment response in RA [7,8]. Candidate-gene studies, although limited to the knowledge of the biological pathways associated to a particular disease or trait, have also been successful in identifying new candidate loci for the response to anti-TNF therapy [9]. One such candidate gene is *FCGR2A*, encoding an Fc receptor mainly expressed in macrophages and dendritic cells [10], and for which there is increasing evidence supporting its association to anti-TNF therapy in RA [11].

Fc receptors for IgG immunoglobulins (FCGRs) are expressed in different immune cells but predominantly in phacogytic cells like macrophages [12,13]. In these cells, FCGRs bind to extracellular IgG immunogloblins, and this binding can lead to either cell activation or repression [13,14,15]. Consequently, the genetic variants that affect the activity of FCGRs [16,17,18,19], could also influence the efficacy of immunoglobulin-based therapies like anti-TNF agents. Infliximab, adalimumab and etanercept are the most commonly used anti-TNF agents in RA, and are characterized by having an IgG1 Fc portion that can bind to FCGRs. Therefore, variations in the Fc binding affinity between the different anti-TNF agents could also influence the response to these biological therapies [20,21,22].

*FCGR2A* (CD32A) SNP rs1801274 is a nonsynonymous polymorphism that leads to an amino acid change at position 131 of the Fc receptor (i.e. R131H). This change in the protein sequence has shown to have important implications in the binding of the receptor to different IgG subclasses [23,24]. Consequently, rs1801274 is a strong candidate for influencing the response to IgG-based treatments, like anti-TNF agents. There is increasing evidence that variation at this SNP is associated with a differential response to anti-TNF therapy in RA [11,25]. Importantly, there is recent evidence that the association between *FCGR2A* and the clinical response in RA could be dependent on the type of anti-TNF agent, with a significant association in patients treated with infliximab [25,26] and a lack of association on etanercept-treated patients [26,27]. Despite the increasing evidence of a strong and differential genetic background associated with patients positive for anti-cyclic citrullinated protein antibodies (anti-CCP, ~70–80% of patients) [28,29,30], very few pharmacogenetic studies in RA have evaluated testing for association in this subgroup of patients. If confirmed, this drug specific associations would be of major relevance for RA. First, it would allow the identification of biological pathways that are specifically targeted by each anti-TNF agent, and secondly, it could lead to the development of new and more specific therapies and finally improve treatment personalization in RA.

The first objective of this study was to validate the association between *FCGR2A* and the clinical response to the main anti-TNF agents infliximab, adalimumab and etanercept. Next, we hypothesized that patients positive for anti-CCP antibodies could show stronger genetic associations to drug response. Also, we hypothesized that analyzing the gene expression correlation of *FCGR2A* in a crucial cell type in RA, synovial fluid macrophage, we could identify new candidate genes associated with anti-TNF response. Using a cohort of well-characterized RA patients we have been able to validate and further characterize *FCGR2A* association, as well as identify new candidate genes for anti-TNF response in RA.

## Materials and Methods

### Study population

A total of 348 RA patients that had received an anti-TNF therapy (infliximab, etanercept or adalimumab) as their first biological treatment, were included in the present study. This patient cohort was collected as part of the Immune-Mediated Inflammatory Disease Consortium (IMIDC) [9], which includes a network of rheumatology departments from 12 university hospitals from Spain. All patients fulfilled the 1987 American College of Rheumatology classification criteria for RA [31] and had >2 years of follow-up since diagnosis. All recruited individuals had an erosive disease defined as 1 ≥ erosions in, at least, 2 joint groups in hands and/or feet. Only RA patients naïve to biologic therapies were included in this study. Patients were Caucasian European born in Spain and with all four grandparents also born in Spain.

Informed consent was obtained from all participants and protocols were reviewed and approved by local institutional review boards. The present study was conducted according to the Declaration of Helsinki principles.

The response to anti-TNF treatment was measured at week 12 following the EULAR treatment response criteria [32]. For all patients, the DAS28 activity score [33] was measured at baseline and after 12 weeks of anti-TNF treatment. According to the change in the DAS28 score and the endpoint DAS28, patients were categorized into good, moderate and none responders. As described previously, EULAR good and moderate responders were combined into a single anti-TNF responder group [9].

### *FCGR2A* correlated genes in synovial RA macrophages

The NCBI Gene Expression Omnibus database of microarray data [34] was used to identify previous studies analyzing the gene expression profiles of macrophages obtained from synovial fluid from rheumatoid arthritis patients. Using the terms "rheumatoid arthritis + macrophage + synovial fluid" we found three different datasets (GSE49604, GSE11575 and GSE10500). From these, we selected the gene expression profiles from RA CD14+ synovial macrophages obtained by positive selection (i.e. GSE49604 and GSE10500). The gene expression profiles from RA macrophages generated by *in vitro* differentiation of blood monocytes (GSE11575 dataset) was not considered in this study. The selected gene expression data was processed and analyzed using the R statistical software [35] and the Bioconductor repository packages (www.bioconductor.org). The details on dataset selection, data preprocessing and correlation analyses are given in the Supporting Information (S1 Protocol). The correlation between *FCGR2A* gene expression and the genes expressed in synovial fluid macrophages was analyzed using the statistical test based on Pearson's product moment correlation. A significance threshold of α = 0.001 was considered to select the genes most strongly correlated with *FCGR2A*.

### DNA collection, SNP selection and genotyping

Whole blood samples were obtained from 348 RA patients and genomic DNA was extracted using the Chemagic Magnetic Separation Module I (Perkin Elmer, US). The *FCGR2A* polymorphism previously associated with the response to anti-TNF therapy, SNP rs1801274 (*FCGR2A-R131H*), was analyzed in the cohort of RA patients using the TaqMan Real-Time PCR platform (Life Technologies, US) with the C___9077561_20 predesigned assay.

Tag SNPs for *DHX32* and *RGS12* genes were selected using the genetic information from the Caucasian European cohort (CEU) sequenced in the 1,000 Genomes Project (1KGP) [36]. Briefly, the dense SNP genotype generated from the 1KGP data from the sequences of the two genes +/- 5 kb was downloaded. Using the Haploview genetic analysis tool (v4.2) [37] we identified the most relevant haplotype blocks in each gene and we selected the corresponding tagSNPs. Additional details on the haplotype analysis and tagSNP selection are given in the Supplementary Material (S1 Protocol). For *DHX32* association analysis we selected tagSNP rs12356233 (chromosome 10, pb 127,534,930), and for *RGS12* locus we selected tagSNP rs2857859 (pb 3,322,140) and rs4690093 (pb 3,412,196). Genotyping was performed with the TaqMan RTCR technology (Life Technologies, US) using predesigned assays C__31490226_30, C__26934339_10 and C__11283507_10 for rs12356233, rs2857859 and rs4690093 genotyping, respectively.

For all Taqman analyses RTPCR thermal conditions were as follows: 50ºC for 2 min and 95ºC for 10 min, followed by 40 cycles of 92ºC for 15 s and 60ºC for 1 min. The PCR assay and point fluorescent readings were performed using an ABI PRISM 7900HT sequence detection system (Life Technologies, USA). Genotyping error was determined by re-genotyping 20% of the patients (<1% genotyping error).

### Statistical analysis

The association of the *FCGR2A* SNP rs1801274 with treatment response was performed using Fisher’s exact test. Given that the association between *DHX3* and *RGS12* SNPs and response to anti-TNF therapy was novel, we used the allelic chi-square test [38]. All statistical tests were performed using the R statistical software version 3.0.1 (www.R-project.org).

## Results

### Study population

A total of 348 RA patients treated with anti-TNF agents were included. The patients had a mean (±SD) age at diagnosis of 43.5 years (±12.6), and an average disease duration of 10.2 years (±8.3). From these, 126 patients had been treated with infliximab, 95 with adalimumab and 127 with etanercept. The complete clinical features of the patient cohort are shown in Table 1.

**Table 1:** Epidemiological and Clinical Features of the Patient Study Cohort.

Variable	All (n = 348)	Infliximab (n = 126)	Adalimumab (n = 95)	Etanercept (n = 127)
Female, n (%)	287 (82.4)	107 (84.9)	76 (80.0)	104 (81.8)
Age at diagnosis (years)	43.5 ± 12.6	43.1 ± 11.6	45.9 ± 12.6	42.2 ± 13.3
Disease duration (years)	10.2 ± 8.3	10.1 ± 6.7	9.7 ± 9.1	10.8 ± 9.1
RF (+), n (%)	270 (77.8)	96 (76.1)	72 (76.6)	102 (80.3)
Anti-CCP (+), n (%)	261 (77.9)	87 (72.5)	79 (85.8)	95 (77.2)
Erosions, n (%)	314 (90.2)	115 (91.3)	85 (89.5)	114 (89.7)
Smokers, n (%)	148 (42.7)	52 (41.6)	43 (45.2)	43 (45.7)
Previous DMARDs	2.7 ± 1.7	3.1 ± 1.7	2.0 ± 1.4	2.7 ± 1.6
Concomitant corticoids, n (%)	182 (52.3)	64 (50.8)	49(51.6)	69 (54.3)
DAS28 (mean+/-SD), baseline	5.6 ± 1.1	5.6 ± 1.1	5.3 ± 1.0	5.7 ± 1.2
DAS28 (mean+/-SD), 12 weeks	3.9 ± 1.4	4.3 ± 1.4	3.5 ± 1.2	3.9 ± 1.5
ΔDAS28 (mean+/-SD)	1.6 ± 1.3	1.4 ± 1.3	1.7 ± 1.2	1.7 ± 1.4
EULAR Responders, n(%)	261(75%)	88 (70%)	76 (80%)	97 (76%)
EULAR Non-Responders, n(%)	87 (25%)	38 (30%)	19 (20%)	30 (24%)

Except where indicated otherwise, values are the mean ±SD. RF: rheumatoid factor; Anti-CCP: anti-citrullinated protein antibodies; DMARDs: disease-modifying antirheumatic drugs; ΔDAS28; delta DAS28 (DAS28 baseline—DAS28 endpoint); EULAR: European League Against Rheumatism response, where Good and Moderate EULAR responders were merged into a single Responder category.

### Association of *FCGR2A* with anti-TNF response

Comparing *FCGR2A* genotype frequencies between the global cohort of anti-TNF therapy responders and non-responders, we found no significant association (*P* = 0.15, Table 2). When analyzing the association within each different anti-TNF agent, we found a statistically significant association between *FCGR2A* and the clinical response to adalimumab (*P* = 0.022, Table 2). In infliximab-treated patients, we did not observe a statistically significant association (*P* = 0.11), while in etanercept-treated patients we found no evidence of association with *FCGR2A* SNP (*P* = 0.96).

**Table 2:** Genotype frequencies of the *FCGR2A* polymorphism rs1801274 according to the clinical response at week 12.

Anti-TNF agent	Genotype	Responders n(%)	Non-responders n(%)	P-value^a^	OR(95%CI)
All (n = 348)					
	AA	67 (25.7)	25 (28.7)	-	-
	AG	143 (54.8)	38 (43.7)	-	-
	GG	51 (19.5)	24 (27.6)	0.15	1.1(0.78–1.56)
Infliximab (n = 126)					
	AA	23 (26.1)	16 (42.1)	-	-
	AG	49 (55.7)	14 (36.8)	-	-
	GG	16 (18.2)	8 (21.1)	0.11	0.76(0.44–1.32)
Adalimumab (n = 95)					
	AA	21 (27.6)	3 (15.8)	-	-
	AG	40 (52.6)	6 (31.6)	-	-
	GG	15 (19.7)	10 (52.6)	0.022	2.54(1.19–5.4)
Etanercept (n = 127)					
	AA	23 (23.7)	6 (20)	-	-
	AG	54 (55.7)	18 (60)	-	-
	GG	20 (20.6)	6 (20)	0.96	1.06(0.6–1.9)

^a^Fisher's exact test; OR: Odds ratio using allele G as reference.

RA patients that are positive for anti-cyclic citrullinated protein antibodies (anti-CCP), have shown to have a differential and much stronger genetic background compared to anti-CCP negative patients [28,29,30]. Based on this observation, we explored the *FCGR2A* association with the response to anti-TNF therapy in the anti-CCP positive group of patients only (78% in our cohort) (Table 3). Analyzing each treatment separately, we found that *FCGR2A* SNP was significantly associated to the response of both adalimumab and infliximab (*P* = 0.047 and *P* = 0.035, respectively). By contrast, we found no association between *FCGR2A* and the clinical response to etanercept (*P* = 1).

**Table 3:** Genotype frequencies of the *FCGR2A* polymorphism rs1801274 in anti-CCP positive RA patients according to the clinical response at week 12.

Anti-TNF agent	Genotype	Responders n(%)	Non-responders n(%)	P-value^a^	OR (95%CI)
All (n = 261)					
	AA	52 (26.8)	21 (31.3)	-	-
	AG	104 (53.6)	26 (38.8)	-	-
	GG	38 (19.6)	20 (29.9)	0.079	1.12(0.76–1.66)
Infliximab (n = 87)					
	AA	15 (24.6)	13 (50)	-	-
	AG	36 (59)	8(30.8)	-	-
	GG	10 (16.4)	5 (19.2)	0.035	0.62(0.32–1.22)
Adalimumab (n = 79)					
	AA	18 (30)	3(15.8)	-	-
	AG	29 (48.3)	6 (31.6)	-	-
	GG	13 (21.7)	10 (52.6)	0.047	2.56(1.18–5.54)
Etanercept (n = 95)					
	AA	19 (26)	5(22.7)	-	-
	AG	39 (53.4)	12 (54.5)	-	-
	GG	15 (20.5)	5 (22.7)	1	1.12(0.57–2.19)

^a^Fisher's exact test; ANTI-CCP: anti-citrullinated protein antibodies; OR: Odds ratio using allele G as reference.

### Identification and analysis of *FCGR2A* functionally-related genes

We identified two microarray-based studies in RA analyzing the transcriptome of synovial macrophages (*GSE49604* and *GSE1050*, n = 8 samples each). In both studies, synovial macrophages were isolated from the synovial fluid by positive CD14+ selection. In each study we found one gene showing a consistent correlation with *FCGR2A* (*P* < 0.001). In GSE49604 study we found *DEAH (Asp-Glu-Ala-His) box polypeptide 32* gene (*DHX32*) levels to be strongly associated to *FCGR2A* gene expression, while in *GSE1050* RA study we found *regulator of G-protein signaling 12* gene (*RGS12*) to correlate significantly with *FCGR2A* levels (S1 Fig). *DH32* was found to positively correlate with *FCGR2A* (average r^2^ = 0.93) and *RGS12* to correlate negatively with *FCGR2A* expression (average r^2^ = -0.96).

For each functionally-related gene we selected tagSNPs and performed an association test with the clinical response to anti-TNF therapy in the RA patient cohort (Table 4). Analyzing all anti-TNF agents together, we found no association between *DHX32* or *RGS12* SNPs with the clinical response at week 12. However, when analyzing each treatment separately, we found a significant association between *DHX32* SNP rs12356233 with the response to adalimumab (corrected *P* = 0.019). We also found a nominally significant association between *RGS12* SNP rs4690093 and the response to adalimumab (uncorrected *P* = 0.040), but this was no longer significant after multiple test correction. When analyzing the anti-CCP positive group of RA patients (Table 5), rs12356233 was still still significantly associated with adalimumab response (corrected *P =* 0.028), and *RGS12* SNP rs4690093 was also associated at the nominal level only (*P* = 0.044). Importantly, in this group of RA patients we also found a nominally significant association between *RGS12* SNP rs2857859 and the response to infliximab (uncorrected *P* = 0.042). This association was not significant after multiple test correction.

**Table 4:** *DHX32* and *RGS12* association with clinical response in RA patients according to anti-TNF therapy.

				All anti-TNF		Infliximab		Adalimumab		Etanercept	
SNP (gene)	Minor Allele	Major Allele	MAF	OR(95%CI)	*P*	OR(95%CI)	*P*	OR(95%CI)	*P*	OR(95%CI)	*P*
rs12356233 (*DHX32*)	*G*	*A*	0.39	1.01(0.71–1.43)	0.96	0.65(0.37–1.15)	0.14	2.7(1.3–5.61)	0.0064*	0.85(0.47–1.56)	0.61
rs2857859 (*RGS12*)	*T*	*C*	0.3	0.87(0.59–1.28)	0.48	0.8(0.43–1.48)	0.48	0.98(0.45–2.15)	0.96	0.89(0.47–1.71)	0.73
rs4690093 (*RGS12*)	*G*	*A*	0.29	0.75(0.51–1.12)	0.16	0.95(0.52–1.71)	0.85	0.4(0.17–0.98)	0.04	0.88(0.44–1.76)	0.71

MAF: minor allele frequency in all RA patients; OR: odds ratio; CI: odds ratio confidence interval; *P*: nominal significance in association test with clinical response at week 12.

*significant (P<0.05) after multiple test correction.

**Table 5:** *DHX32* and *RGS12* association with clinical response in anti-CCP positive RA patients according to anti-TNF therapy.

				All anti-TNF		Infliximab		Adalimumab		Etanercept	
SNP (gene)	Minor Allele	Major Allele	MAF	OR(95%CI)	P	OR(95%CI)	P	OR(95%CI)	P	OR(95%CI)	P
rs12356233 (*DHX32*)	*G*	*A*	0.39	1.09(0.73–1.64)	0.66	0.85(0.42–1.7)	0.64	2.65(1.25–5.6)	0.0095*	0.7(0.35–1.42)	0.32
rs2857859 (*RGS12*)	*T*	*C*	0.29	0.64(0.4–1.03)	0.062	0.4(0.17–0.99)	0.042	1.01(0.45–2.25)	0.99	0.68(0.31–1.48)	0.33
rs4690093 (*RGS12*)	*G*	*A*	0.3	0.85(0.55–1.32)	0.46	1.13(0.57–2.24)	0.73	0.4(0.16–0.99)	0.049	1.06(0.48–2.31)	0.89

MAF: minor allele frequency in all anti-CCP RA patients; OR: odds ratio; CI: odds ratio confidence interval; *P*: nominal significance in association test with clinical response at week 12.

*significant (P<0.05) after multiple test correction.

## Discussion

Anti-TNF agents have been a major success in RA treatment, significantly improving the prognosis of many patients. There is, however, a group of RA patients that does not respond significantly to this therapeutic approach. Consequently, there is a major need to identify biomarkers that can help predict anti-TNF response and therefore guide anti-TNF therapy. In the present study we have used a cohort of RA patients from the Spanish population to validate the association between *FCGR2A* and anti-TNF agents infliximab, adalimumab and etanercept. Also, we have identified the genes most strongly correlated with *FCGR2A* expression in synovial fluid macrophages from RA studies, and we have found an association between these new, functionally-related genes with the response to anti-TNF therapy.

In our patient cohort we have found, for the first time, that *FCGR2A* is significantly associated with the response to infliximab in anti-CCP positive RA patients. Anti-CCP antibodies are known to bind to citrullinated antigens expressed in the synovial joint like filaggrin, and lead to the formation of immune complexes which are powerful activators of the immune response [39]. FCGR2A receptor has been shown to be key in the internalization of immune complexes by phagocytic cells and consequently, could influence citrullinated peptide presentation by HLA proteins and the subsequent activation of autorreactive T cells [40]. In this context, differences in the affinity of FCGR2A for citrullinated immune complexes could be counteracting the beneficial effect of ADCC by inducing a major response to citrullinated autoantigens. The results of this study, therefore, support the importance of anti-CCP antibodies in the FCGR2A-mediated response to anti-TNF agents.

The genetic analysis of genes functionally related to *FCGR2A* in synovial macrophages from RA patients, has lead to the identification of two new genes, *DHX32* and *RGS12* with the response to anti-TNF therapy. *DHX32* gene encodes for a putative RNA helicase, and has been associated with lymphocyte differentiation and activation [41,42,43,44]. Importantly, RNA helicases have also shown to be important in innate immunity inactivation of viral RNA [45], and there is an increasing evidence that deregulation in this pathogen-sensing pathways could contribute to the development of autoimmunity, including RA [46,47,48,49]. Together, these results support the contribution of innate immunity in the differential response to anti-TNF therapies.

*RGS12* encodes for a member of the 'regulator of G protein signaling' gene family although its biological still role remains to be characterized. Transcriptomic analysis on mouse monocyte progenitor cells activated with Receptor Activator of NF-kappa-B (RANKL) have shown a high induction of *RGS12* gene [50]. *RGS12* was shown to be highly and specifically expressed in human osteoclasts, and the inhibition of RGS12 in mouse monocyte cultures impaired RANKL-mediated differentiation to osteoclasts. These results suggest that *RGS12* could participate in the macrophage to osteoclast transition, and therefore contribute to bone erosion and disease activity in RA.

Like most genetic studies for drug response, the present study has limitations. Issues like the uncertainty on the true genetic model underlying *FCGR2A* association, the definition of clinical response or the presence of environmental or genetic interactions could have reduced the statistical power of the study. For example, analyzing only those patients with a more extreme clinical response (i.e. EULAR good vs. none responders), only the *FCGR2A* association with adalimumab in all patients is still significant (S1 and S2 Tables). In this case, discarding the EULAR moderate group of patients, which is also the most frequent type of response (i.e. >40% of anti-TNF treated patients), clearly reduces the statistical power to identify the genetic associations. Also, from the observed genotype *FCGR2A* frequencies, there is no obvious gene dosage effect. However, in a complex trait like anti-TNF response there is likely to be a polygenic component, with multiple risk genes of moderate to low effect size where genetic models are more difficult to characterize. The presence of interaction effects can also limit our ability to fully characterize the present pharmacogenetic association. In anti-CCP positive RA patients, smoking has shown to strongly interact with *HLA-DRB1* genotypes in the risk to develop the disease [51]. In our cohort of anti-CCP positive RA patients we did not find a significant interaction between *FCGR2A* and smoking status (data not shown). Finally, the identification of new anti-TNF response genes using gene expression profiles is clearly subject to isolating the relevant cell type where the gene is expressed as well as the methodological approach used to characterize the gene expression profiles. However, the association of genetic variants in *DHX32* and *RGS12* loci associated with anti-TNF response, strongly supports the role of these two new genes in RA pathophysiology.

In the present study, we confirm the association between *FCGR2A* and the response to anti-TNF therapy. Also, using transcriptomic data from synovial fluid macrophages from RA studies, we have identified two genes, *DHX32* and *RGS12*, strongly correlated with *FCGR2A* expression. Analyzing variants in these two new candidate genes, we have found new genetic associations with treatment response. The results of this study demonstrate a complex genetic basis for anti-TNF response and are an important advance in the understanding of the molecular mechanisms associated with the response to these therapies.

## Supporting Information

S1 Fig Significantly correlated genes with *FCGR2A* in synovial macrophages. Plots of the two probes measuring *FCGR2A* expression in SMPA and SMPB microarray studies with respect to the most significantly correlated genes (P < 0.001, *DHX32* in SMPA and RGS12 in SMPB). The red line depicts the linear regression model of each gene against *FCGR2A* expression.
(TIFF)

S1 Protocol Includes the detailed description of the synovial macrophage microarray dataset selection, data preprocessing and association analysis. It also includes details on the methodology used to select tagSNPs for each gene, *DHX32* and *RGS12*, functionally associated with *FCGR2A*.
(DOCX)

S1 Table *FCGR2A* polymorphism frequencies according to the EULAR extreme clinical response. (DOCX)

S2 Table *FCGR2A* polymorphism frequencies in anti-CCP positive RA patients according to the EULAR clinical extreme response. (DOCX)
