# Supplementary figures and images for: Variation at FCGR2A and Functionally Related Genes Is Associated with the Response to Anti-TNF Therapy in Rheumatoid Arthritis
Source: PLoS One. 2015 Apr 7;10(4):e0122088. doi: 10.1371/journal.pone.0122088 (PMC4388501; doi:10.1371/journal.pone.0122088)

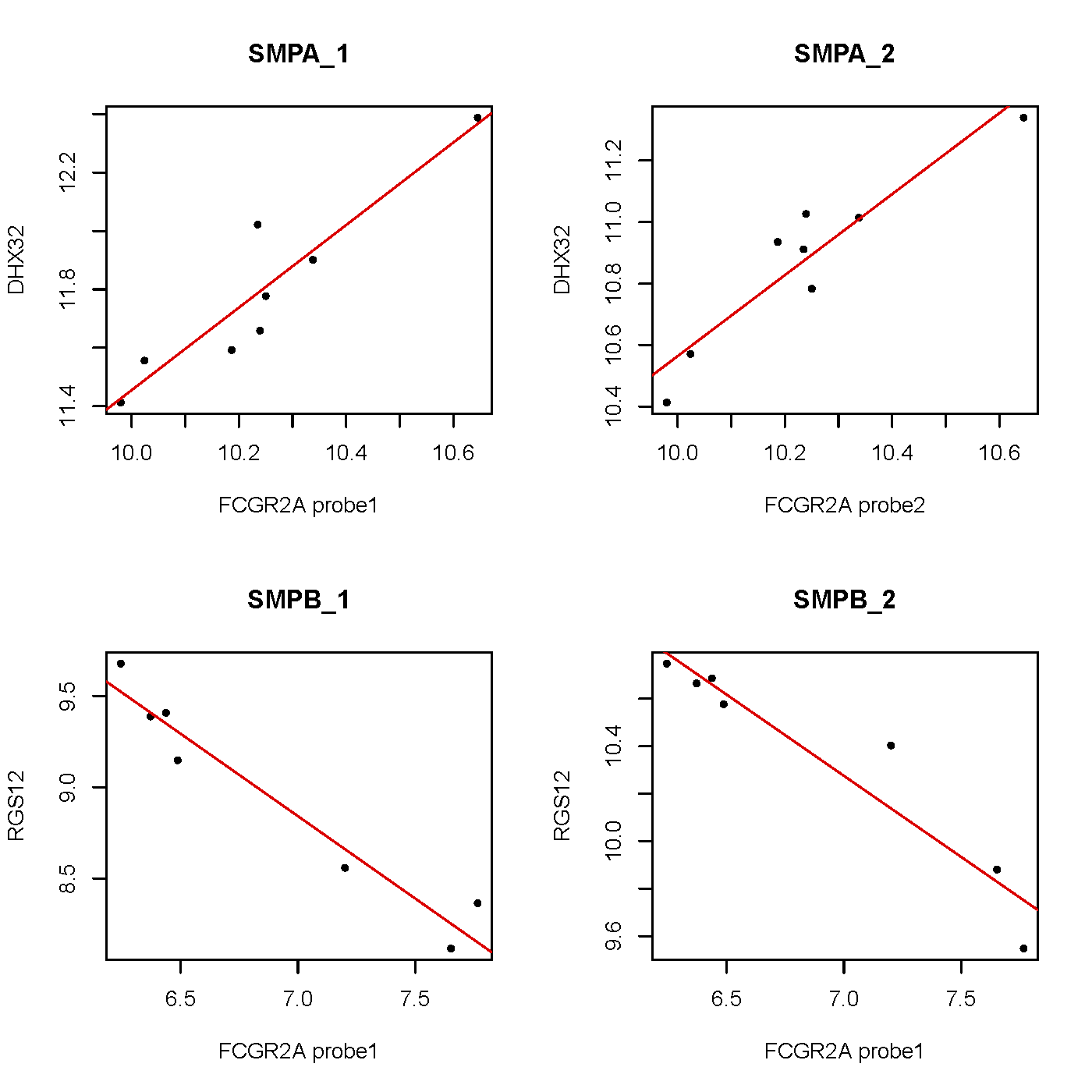

Supplement: S1 Fig — Plots of the two probes measuring FCGR2A expression in SMPA and SMPB microarray studies with respect to the most significantly correlated genes (P < 0.001, DHX32 in SMPA and RGS12 in SMPB). The red line depicts the linear regression model of each gene against FCGR2A expression. (TIFF) [file pone.0122088.s001.tiff]
