# Supplementary material for: Variation at FCGR2A and Functionally Related Genes Is Associated with the Response to Anti-TNF Therapy in Rheumatoid Arthritis
Source: PLoS One. 2015 Apr 7;10(4):e0122088. doi: 10.1371/journal.pone.0122088 (PMC4388501; doi:10.1371/journal.pone.0122088)
Supplement: S1 Protocol — It also includes details on the methodology used to select tagSNPs for each gene, DHX32 and RGS12, functionally associated with FCGR2A. (DOCX) [file pone.0122088.s002.docx]

# Microarray data analysis

## Dataset selection

Whole genome gene expression analyses on macrophage cells isolated from synovial fluid of rheumatoid arthritis patients (RA) were selected from the Gene Expression Omnibus microarray repository (http://www.ncbi.nlm.nih.gov/gds). Querying by the terms "macrophage + rheumatoid arthritis + synovial fluid" we found three different datasets: *GSE49604*, *GSE10500* and *GSE11575*. From these, gene expression profiles generated from *in vitro* activated whole blood monocytes were discarded, and only transcriptomes obtained from synovial fluid macrophages were selected. Accordingly, the analyzed gene expression profiles were:

- Synovial macrophage profile A) *GSE49604*: CD14+ macrophages isolated from synovial fluid of 6 RA patients were isolated by positive selection using magnetic beads. In controls (n=2), CD14+ monocytes were obtained from peripheral blood using magnetic beads, and were cultured for 24 hours in the presence of M-CSF [[1](#_ENREF_1)]. After RNA extraction gene expression profiles were generated using Illumina (Illumina, San Diego, USA) HumanRef-8 WG-DASL v3.0 array (http://www.illumina.com/).

- Synovial macrophage profile B) *GSE10500*: CD14+ macrophages isolated from synovial fluid of 5 RA patients were isolated by positive selection using magnetic beads. In controls (n=3), CD14+ monocytes were obtained from peripheral blood using magnetic beads, and were cultured for48 hours in the presence of M-CSF [[2](#_ENREF_2)] .After RNA extraction gene expression profiles were generated using the Affymetrix (Affymetrix, Santa Clara, USA) Human Genome U95 Version 2 Array (http://www.affymetrix.com/support/technical/byproduct.affx?product=hgu95).

## Data preprocessing

All statistical analyses were performed using the R statistical software language (www.cran.org) and the Bioconductor repository of libraries specialized in the analysis of genomic data (www.bioconductor.org). Accordingly, the microarray data was downloaded using the "GEOquery" package. Synovial macrophage profile A was log2 transformed and normalized using the quantile normalization method implemented in "preprocessCore" package. Profile B was already transformed and normalized so no preprocessing method was applied.

In each microarray platform 2 probes were identified that measured the gene expression of FCGR2A:

- Affymetrix HG_U95Av2: probes *37688_f_at* and *37689_s_at*.

- Illumina HumanRef-8: probes *ILMN_2289849* and *ILMN_1706523*.

Examining the expression levels of the 4 probes in all the samples we found 1 sample (GSM265020) to have an clearly outlying expression level (i.e. >2 standard deviations from mean) and was removed from the analysis (Supplementary Figure S2).

**Figure S2. Gene expression values of FCGR2A probes.** Boxplots of gene expression values of the two *FCGR2A* probes for each synovial macrophage microarray study. SMPA: synovial macrophage profile A; SMPB: synovial macrophage profile B. In SMPB one sample has an outlying value (>2 SD of mean, black dot) and was removed from the analysis.

## Association analysis

The correlation between the probes measuring *FCGR2A* expression and the remaining genes in the genome was analyzed using the test for correlation based on Pearson's product moment correlation. In order to select those genes showing a higher evidence of correlation with *FCGR2A* expression, we used a significance threshold of α = 0.001 in for the correlation with the two probes measuring the gene expression levels.

In the Synovial Macrophage Profile A we found that the probe ILMN_1713688 measuring the expression of *DEAH (Asp-Glu-Ala-His) box polypeptide 32* gene (*DEX32*) was significantly correlated with *FCGR2A* gene expression. In the Synovial Macrophage Profile B we found that probe *32580_at* measuring the expression of *regulator of G-protein signaling 2* gene (*RGS12*) was also significantly correlated with the gene expression of *FCGR2A* (S1 Fig.).

# TagSNP selection for *FCGR2A* related genes

Using the high density genotype data obtained by the 1KG sequencing project we selected the markers covering most of the common variability in each gene. First the SNP variation data for the Caucasian European CEU Hapmap population was downloaded using the 1KG genome browser (http://browser.1000genomes.org/index.html). The SNP variants within the RefSeq gene coordinates +/- 5 kilobases (DHX32: chromosome 10 pb 127,519,909 to 127,574,884 and RGS12: chromosome 4 pb 3,310,874 to 3,446,640) and were downloaded and analyzed using the Haploview haplotype analysis software.

After filtering for low minor allele frequency variants (MAF < 0.10) we selected we estimated the haplotype blocks present at teach locus using the method by Gabriel et al. [[3](#_ENREF_3)]. *DHX3* was found to be characterized by a single haplotype block while *RGS12* had two major haplotype blocks in this locus (**Figures S3 A** and **B**). For each haplotype block we used Happloview to select the best tagging SNP. For *DHX3* association analysis we selected tagSNP rs12356233 (pb 127,534,930) and for *RGS12* analysis we selected tagSNPs rs2857859 (pb 3,322,140) and (pb 3,412,196).

**Figure S3. Haplotype block estimation for *DHX32* and *RGS12* genes.** Using dense genetic data obtained from the 1KG project on CEU samples, we determined the haplotype blocks for the two candidate genes for anti-TNF response in RA (**A**) DHX32 and (**B**) RGS12. *DHX32* is a small gene (~45kb) and is characterized by a single haplotype block *RGS12* is a larger gene (~126kb) and has two major haplotype blocks that cover most of the genetic region.
