# Supplementary material for: Variation at FCGR2A and Functionally Related Genes Is Associated with the Response to Anti-TNF Therapy in Rheumatoid Arthritis
Source: PLoS One. 2015 Apr 7;10(4):e0122088. doi: 10.1371/journal.pone.0122088 (PMC4388501; doi:10.1371/journal.pone.0122088)
Supplement: S1 Table — (DOCX) [file pone.0122088.s003.docx]

| **Supplementary Table 1.** *FCGR2A* polymorphism frequencies according to the EULAR extreme clinical response**.** | | | | | |
| --- | --- | --- | --- | --- | --- |
|  |  |  |  |  |  |
| **Anti-TNF agent** | **EULAR Good n(%)** | | **EULAR None n(%)** | | **P-value** |
| *All (n=202)* |  |  |  |  |  |
| *AA* | 33 (28.7) | | 25 (28.7) | | 0.57 |
| *AG* | 57 (49.6) | | 38 (43.7) | |  |
| *GG* | 25 (21.7) | | 24 (27.6) | |  |
| *Infliximab (n=67)* |  |  |  |  |  |
| *AA* | 8 (27.6) | | 16 (42.1) | | 0.49 |
| *AG* | 13 (44.8) | | 14 (36.8) | |  |
| *GG* | 8 (27.6) | | 8 (21.1) | |  |
| *Adalimumab (n=60)* |  |  |  |  |  |
| *AA* | 12 (29.3) | | 3 (15.8) | | **0.047** |
| *AG* | 21 (51.2) | | 6 (31.6) | |  |
| *GG* | 8 (19.5) | | 10 (52.6) | |  |
| *Etanercept (n=75)* |  |  |  |  |  |
| *AA* | 13 (28.9) | | 6 (20) | | 0.66 |
| *AG* | 23 (51.1) | | 18 (60) | |  |
| *GG* | 9 (20.0) | | 6 (20) | |  |
| ^a^ Fisher's exact test. | | | | | |
|  |  |  |  |  |  |
