# Supplementary material for: Variation at FCGR2A and Functionally Related Genes Is Associated with the Response to Anti-TNF Therapy in Rheumatoid Arthritis
Source: PLoS One. 2015 Apr 7;10(4):e0122088. doi: 10.1371/journal.pone.0122088 (PMC4388501; doi:10.1371/journal.pone.0122088)
Supplement: S2 Table — (DOCX) [file pone.0122088.s004.docx]

| **Supplementary Table 2.** *FCGR2A* polymorphism frequencies in anti-CCP positive RA patients according to the EULAR clinical extreme response. | | | | | |
| --- | --- | --- | --- | --- | --- |
|  |  |  |  |  |  |
| **Anti-TNF agent** | **EULAR Good n(%)** | | **EULAR None n(%)** | | **P-value** |
| *All (n=156)* |  |  |  |  |  |
| *AA* | 28 (31.5) | | 21 (31.3) | | 0.43 |
| *AG* | 42 (47.2) | | 26 (38.8) | |  |
| *GG* | 19 (21.3) | | 20 (29.9) | |  |
| *Infliximab (n=50)* |  |  |  |  |  |
| *AA* | 6 (25) | | 13 (50) | | 0.21 |
| *AG* | 12 (50) | | 8(30.8) | |  |
| *GG* | 6 (25) | | 5 (19.2) | |  |
| *Adalimumab (n=51)* |  |  |  |  |  |
| *AA* | 11 (34.4) | | 3(15.8) | | **0.072** |
| *AG* | 14 (43.8) | | 6 (31.6) | |  |
| *GG* | 7 (21.9) | | 10 (52.6) | |  |
| *Etanercept (n=95)* |  |  |  |  |  |
| *AA* | 11 (33.3) | | 5(22.7) | | 0.76 |
| *AG* | 16 (48.5) | | 12 (54.5) | |  |
| *GG* | 6 (18.2) | | 5 (22.7) | |  |
| ^a^ Fisher's exact test; ANTI-CCP: anti-citrullinated protein antibodies. | | | | | |
|  |  |  |  |  |  |
